# Lupus Erythematous Profundus: An Unusual Manifestation in the Otolaryngological Setting

**DOI:** 10.7759/cureus.46402

**Published:** 2023-10-03

**Authors:** Clarisse Chu, Leonard Soh, Sze Hwa Tan, Tee Sin Lee, Ian Loh

**Affiliations:** 1 Otolaryngology, Changi General Hospital, Singapore, SGP; 2 Pathology, Changi General Hospital, Singapore, SGP

**Keywords:** abscess, infection, diagnostic dilemma, otolaryngology, lupus profundus

## Abstract

Lupus erythematous profoundus (LEP) is an uncommon manifestation of chronic lupus erythematous (CLE) involving inflammation of the subcutaneous fat and deep dermis. It is rarely seen in the otolaryngological practice. We describe a case of a 33-year-old female who presented with bilateral acute onset cheek swelling, which led to the unexpected diagnosis of LEP. We describe the diagnostic pitfalls that may potentially bias the surgeon towards the management of such patients.

## Introduction

Lupus erythematosus profundus (LEP) is a rare clinical variant of chronic lupus erythematosus (CLE) [1]. It involves the subcutaneous fat and deep dermis, presenting with indurated plaques and/or tender subcutaneous nodules that heal with scarring and lipoatrophy [2]. These lesions are usually asymmetrically distributed, affecting the face, proximal extremities, neck, buttocks, and chest [3]. LEP tends to occur on its own, but may sometimes coexist with systemic lupus erythematosus (SLE) and discoid lupus erythematosus (DLE) [4].

We present an unusual case of bilateral acute onset cheek swelling, which led to a diagnosis of LEP.

## Case presentation

**Figure 1:**
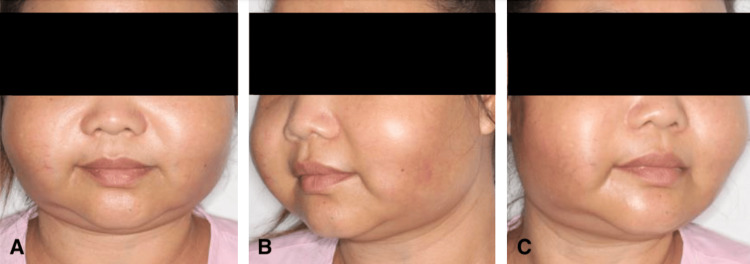
Bilateral diffuse cheek swellings​​​​​​​​​​​​​​

A 33-year-old Burmese female presented to the Otolaryngology Department with progressive bilateral painful cheek swellings (Figure 1). She had been treated with a three-day course of co-amoxiclav by the general practitioner prior to presentation, but sought further medical attention as the cheek swelling continued to increase in size.

The patient denied fever, weight loss, night sweats, malaise, joint pains, rashes, and sicca symptoms. She had no past medical history and was not on any long-term medication. There was no history of trauma, facial injections, or dental procedures, and no family history of autoimmune disease. On physical examination, there were bilateral diffuse cheek swellings, which were tender and firm. There were no overlying skin changes, no erythema or ulceration. There was an associated trismus of 1.5 finger breadths. Oral cavity and dental examination were unremarkable. There was no palpable cervical lymphadenopathy.

**Figure 2:**
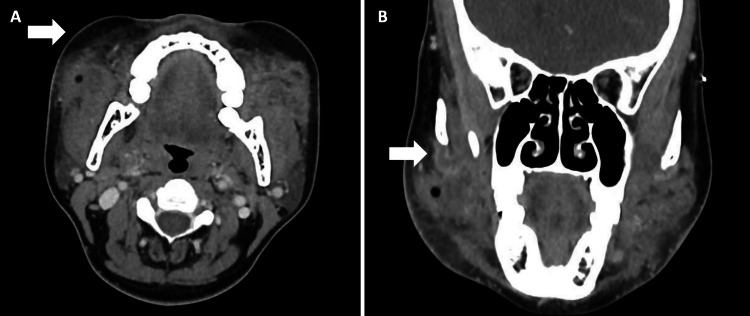
Rim-enhancing collection with internal gas locule (1.8 x 1.6 x 1.8 cm) extending into the intermuscular plane between the right masseter and buccinator muscles

**Figure 3:**
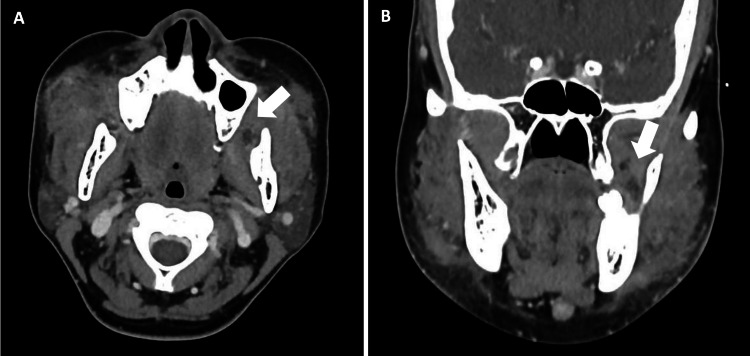
Irregular fluid collection with incomplete rim enhancement (0.9 x 0.8 x 1.2 cm) in the left masticator space

The initial impression was that of a possible bilateral masticator space abscess secondary to melioidosis. Further investigations with computed tomography (CT) scan of the neck with contrast revealed heterogenous soft tissue enhancement with extensive surrounding inflammatory fat stranding centered at the buccal space, with extension to the submandibular and masticator spaces bilaterally. Of note were suggestions of possible early abscesses with rim-enhancing collections with an internal gas locule extending into the intermuscular plane between the right masseter and buccinator muscles (Figure 2) as well as irregular fluid collections in the left masticator space (Figure 3). Enlarged submental and bilateral submandibular lymph nodes were also noted. She was initially treated with intravenous co-amoxiclav, with a view to consider drainage of the abscesses if there was no clinical improvement.

In view of her unusual presentation, an Infectious Diseases opinion was sought. Her clinical presentation was deemed uncharacteristic of atypical infections. Leukocyte count was normal, and inflammatory markers of erythrocyte sedimentation rate (ESR) and C-reactive protein (CRP) were only mildly raised. Blood cultures were negative. Meliodoisis titers were borderline positive (1:16), likely due to meliodoisis being endemic in Burma, and the rest of her clinical picture was not suggestive. There was a borderline positive tuberculosis (TB) spot; however. chest radiograph was unremarkable, suggestive of latent TB as there was no clinical evidence of active TB infection. Hepatitis C antibody screen was false positive, with no detectable viral load. Hepatitis B, human immunodeficiency virus (HIV), and *Brucella* serologies were negative. Autoimmune screen including antinuclear antibody (ANA), anti-double stranded DNA (anti-dsDNA), anti-neutrophil cytoplasmic antibody (ANCA), anti-Ro, anti-La, anti-Scl70, anti-Jo-1, anti-MPO, and anti-PR3 were negative.

**Figure 4:**
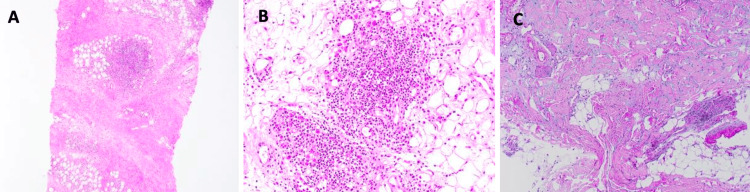
(A) H&E, magnification x40, section shows thickened septa and dense lymphoplasmacytic infiltrate involving the fat lobules with areas of fat necrosis and hyalinization. No marked eosinophilia or areas of storiform fibrosis suggestive of possible IgG4-related disease can be discerned; (B) H&E, magnification x200, higher power demonstrating lymphoplasmacytic infiltrate with the plasma cells containing Russell bodies; (C) Alcian blue/PAS stain, magnification x100, increase in dermal mucin deposition H&E: hematoxylin and eosin; PAS: periodic acid–Schiff

A skin punch biopsy taken over the right cheek, down to the level of the subcutis, showed lobular panniculitis with dense lymphoplasmacytic infiltrate, fat necrosis, and diffuse hyalinization. There was no marked eosinophilia or areas of storiform fibrosis to suggest possible IgG4-related disease (Figure 4).

The patient was co-managed with rheumatology and treated with tapering doses of oral prednisolone over three weeks. On review after initiation of steroid therapy, there was significantly reduced swelling and induration of skin over the cheeks.

## Discussion

LEP is primarily seen in the setting of Dermatology and Rheumatology. Rarely does it present to Otolaryngology, with one case report by Jefferson et al. on LEP presenting as chronic submental masses, initially thought to be sebaceous cysts [5].

Given the acute and dramatic nature of our patient’s presentation, as well as the presence of significant abscesses, the clinician may be skewed towards an infective process. It may bias the surgeon toward a consideration of draining the abscess. However, the bilateral presentation of cheek swelling points towards a more atypical and systemic process. In addition, bilateral abscess formation in such a uniform distribution without a history of previous procedures is also unlikely. In light of the rarity of LEP, an Infectious Diseases opinion was sought. While melioidosis is a consideration in such cases, it can be easily excluded through serological testing and the lack of pulmonary symptoms.

It is important to note that most patients with LEP do not have systemic manifestations of SLE and DLE. Erythema, while often associated with panniculitis, may sometimes be absent, adding to the diagnostic challenge [6]. Serological markers may be nonspecific, thus histopathological confirmation is necessary. A skin punch biopsy is a relatively simple bedside procedure that facilitates the diagnostic procedure. It is only then appropriate treatment can be instituted.

The mechanism by which LEP lesions occur is not well understood; an inflammatory process resulting in hypodermal necrosis may play a role. LEP lesions have been described to develop over areas of trauma, biopsy, and injection sites [7,8]. Especially in acute cases, it may be precipitated by micro-trauma. Kimball et al. described a case of a patient who fell asleep on her desk and bumped her cheek the day before symptom onset [9].^ ^Minor trauma during the course of her daily activities that went unreported could possibly be a trigger for our patient’s presentation.

There is little literature on the imaging features of LEP. Kimball et al. and Vattoth and Curé described CT findings of abnormal fat stranding in the masticator and buccal spaces [9,10]. Razek described soft tissue thickening and infiltration of the cheek [11]. It is interesting to note how the inflammation on our patient’s CT was out of proportion to the swelling on clinical examination. This is also the first report of LEP presenting as early micro-abscesses with partial to complete rim enhancement. It is important to keep this red herring in mind, taking the rest of the clinical picture into account in the diagnosis of LEP over an infective etiology.

## Conclusions

LEP of the cheek can mimic infective processes, particularly when it presents dramatically in the acute setting, with micro-abscesses on CT imaging. Although rare, it is an important differential to consider. Its presentation in the surgical setting may potentially bias the surgeon towards drainage of the abscess, rather than appropriate management with immunosuppressants. Differentials like meliodosis can be excluded through serological tests and confirmation with a simple bedside skin punch biopsy.
